# Higher tax and less work: reverse “Keep up with the Joneses” and rising inequality

**DOI:** 10.1007/s00712-023-00821-2

**Published:** 2023-03-29

**Authors:** Felix FitzRoy, Jim Jin, Michael Nolan

**Affiliations:** 1 School of Economics and Finance, University of St Andrews, St Andrews, KY16 9AL UK; 2 School of Economics and Finance, University of St Andrews, St Andrews, KY16 9AL UK; 3 Faculty of Business, Law and Politics, University of Hull, Hull, HU6 7RX UK

**Keywords:** Income comparison, Maxi-min, Inequality, Unemployment, H240, D630

## Abstract

To counteract excessive effort due to relative income comparison among identical agents, the literature suggests a tax response equal to the negative externality. Assuming a general income distribution, we show that an optimal tax must be higher under a general social welfare function, to not only reduce inefficiency but also inequality. We recommend a practical tax response to stronger comparison – to hold employment constant, which does not require unrealistic information including unobservable comparison. Surprisingly, the tax response will dominate the comparison effect and *reduce* labour supply or reverse “keeping up with the Joneses” on intensive margins, and also reverse the otherwise rising inequality.

**Supplementary Information:**

The online version contains supplementary material available at 10.1007/s00712-023-00821-2.

## Introduction

Income comparison generates a negative externality and leads to extra effort to ‘keep up with the Joneses’ (KUJ). With a general distribution of wages and both intensive and extensive margins of labour supply, we extend the existing literature on tax policy dealing with not only efficiency loss but also increased inequality. We show that an optimal tax should be higher than the negative externality and suggest a practical tax policy response to stronger concern for relative incomes: one that maintains constant employment. This policy does not require unrealistic information about unobservable variables and is valid under general objective functions. It will not only reduce the efficiency loss but also reverse KUJ and rising inequality.

Conspicuous consumption due to affluence, as first criticised by Veblen (1899) and KUJ have become increasingly widespread under the influence of advertising and social media. While subjective wellbeing (SWB) or happiness rose slightly with economic growth in Europe and Japan in 1973–2007 (Stevenson and Wolfers 2008), it has remained largely constant in the west and even declined in the US over the last half century (Easterlin 1974, 1995, 2013, 2021; Easterlin and O’Connor 2020; Kaiser and Vendrik 2018; Rojas 2019), likely due to rising inequality and declining social mobility (Stiglitz 2012, 2019; Piketty 2014; Atkinson 2015). Happiness is found to be negatively related to the income share of the top 1% (Burkhauser et al. 2016) and screen time on social media (Nesi and Prinstein 2015; Twenge et al. 2018; Spitzer 2017; Mujcic and Oswald 2018).

Following pioneering discussions by Duesenberry (1949), Leibenstein (1950), Runciman (1966), Layard (1980), Oswald (1983), and Frank (1985), recent evidence on the negative effects of comparison on SWB has been reviewed by McBride (2001), Clark et al. (2008), and Layard et al. (2010)^1^. It is evident that income comparison leads to ‘keeping up with the Joneses’ (KUJ) (Bowles and Park 2005; Bracha et al. 2015; Goerke and Pannenberg 2013; Pérez-Asenjo 2011), which contributes to persistent long working time, in contrast to Keynes’s (1930) prediction of a 15-hour work week by 2030.

To deal with inefficient income comparison, Boskin and Sheshinski (1978) first recommend tax responses and find that the optimal marginal tax can be close to 100% even under a utilitarian objective, but the precise level depends on unobservable strength of comparison. Assuming identical workers Ljungqvist and Uhlig (2000) obtain an optimal consumption tax equal to the (unobservable) negative externality of comparison. Allowing different agents, Corneo (2002) shows that progressive income taxes can be a Pareto improvement. Tax responses with a representative agent are generalized to various circumstances by Dupor and Liu (2003), Guo (2005) and Mujcic and Frijters (2015). With two types of workers Aronsson and Johansson-Stenman (2008) find that the elegant result of Ljungqvist and Uhlig (2000) remains Pareto efficient if two types of workers have the same marginal rate of substitution^2^, which does not hold in general and not in Ljungqvist and Uhlig (2000) if there were two types. In general, a real economy contains far more than two types of agents, and the optimal tax must depend on how these types are distributed across the population. With specific income distributions and numerical simulation Kanbur and Tuomala (2013), Tuomala (2016), FitzRoy and Nolan (2016) and Slack and Ulph (2018) find different optimal taxes in response to stronger income comparison according to specific income distributions and welfare functions, e.g., maxi-min and utilitarian.

Different types of agents usually have different marginal utilities of consumption. This means that existing policy recommendations, based on only one or two types, could be expected to exhibit significant limitations. We show that an optimal tax rate should always be higher than the externality of income comparison. Needless to say, its precise value depends on income distributions and social welfare functions as well as the strength of comparison, and so is difficult to find. The existing policy recommendations are based on a measurable level of relative income concern and other unobservable variables. As pointed out by Boskin and Sheshinski (1978), an optimal tax is very sensitive to such concern, which is hard to measure. A practical and robust policy response cannot really rely on such knowledge, or on simulation results with specific assumptions. The challenge is to translate qualitative insights into a practical tax policy. It should be applicable to a general income distribution and flexible objectives, independent of relative income comparison and other unobservable variables.

In this paper, we develop a simple model with a general income distribution and unemployment to examine KUJ on both intensive and extensive margins and evaluate the impact of comparison on inequality as well as inefficiency. Following the literature, e.g., Ljungqvist and Uhlig (2000), we assume that tax revenue funds a demogrant or universal basic income (UBI). We first show that, in the absence of a tax response, stronger comparison will not only hurt everyone, but also hurt the lower income earners more, and hence increase inequality. Thus, an optimal tax policy should aim to reduce inequality as well as inefficiency. We then consider the optimal tax first under maxi-min and later under a general welfare function with non-increasing weight assigned to higher income earners. We find that an optimal tax rate is always higher than the negative externality from comparison, instead of being equal as in the previous literature assuming a representative agent. This tax must be sensitive to the income distribution and objective function as well as the strength of comparison.

Instead of a complex tax solution based on unrealistic information we obtain three surprising and practical results: (i) the optimal tax should maintain a fixed employment independent of the strength of comparison, i.e., it should eliminate the KUJ effect on extensive margins; (ii) the optimal tax actually reverses the KUJ effect on intensive margins – individual labour supply *declines*; and (iii) the lower earners will suffer less and inequality declines.

A major novelty here is that our optimal tax should be set to maintain the same level of employment in response to stronger comparison, independent of income distributions and social objectives. Although real-world taxes are rarely optimal, policy makers might reach the best feasible solution after an extended period of trial and error. Thus, it may not be entirely unrealistic to assume that the tax was optimal under the policy maker’s objective before comparison rises. Then, the tax response to stronger comparison should counter its effect by maintaining the same level of employment. It does not require derivation of the optimal tax based on complete knowledge of the economy, including the strength of comparison. The optimal tax will reduce labour supply on intensive margins, i.e., reverse KUJ even though it does not affect employment at extensive margins. Consequently, lower earners suffer less and higher earners more when comparison increases, while the opposite would occur in the absence of a tax response. Therefore, with the tax response inequality will decrease instead of increase when concerns for relative income rise. These policy recommendations seem to be new in terms of less restrictive assumptions and closer to practical implementation. They may contribute to solving or alleviating the KUJ problem.

The model is set out in Sect. 2 below, followed by analysis of the impact of comparison in Sect. 3. The optimal tax responses and policies are obtained in Sect. 4 and conclusions are summarised in Sect. 5.

## A simple model of comparison

Our model follows Ljungqvist and Uhlig (2000), but we assume an economy with a unit population and a continuous distribution of wages $w \in$ [0, *b*]. The distribution function is denoted by $F(w)$ and the density function *f*(*w*). Individual labour supply is *l* ≥ 0. With wage *w* one earns *wl* and receives after-tax earnings (1 – *t*)*wl*. The total tax revenue is distributed to everyone as a demogrant denoted by *B*. The net income of wage earners is (1 – *t*)*wl* + *B*, which is equal to their consumption *c*. Let *y* denote total income or output $\int_{m}^{b} {wlf(w)dw}$, which is equal to total consumption *C*. Total income and consumption are also the average given a unit population and equal to total net income (1 – *t*)*y* + *B* as *B* = *ty*.

Following Johansson-Stenman et al. (2002) the strength of comparison is represented by *β* ∈ (0, 1), which generates a status factor from one’s relative consumption compared to the average, *β*(*c* – *C*), and reduces the importance of own consumption to (1 – *β*)*c*. The sub-utility from consumption is *u*[(1 – *β*)*c* + *β*(*c* – *C*)], where *β* reflects the degree of positionality as frequently assumed in the literature. The comparison *c* – *C* is between one’s net income and the average net income. As (1 – *β*)*c* + *β*(*c*– *C*) = *c* – *βC*, the sub-utility reduces to a simple form of *u*(*c* – *βC*) as assumed by Ljungqvist and Uhlig (2000)^3^.

Again following Ljungqvist and Uhlig (2000), we assume that an individual’s utility is quasi-linear in consumption and leisure as *u*(*c* – *βC*) – *Al*, where *A* > 0 and *u*(*c* – *βC*) is a concave power function. As explained by Ljungqvist and Uhlig (2000), this function implies no comparison in leisure, only in consumption. The linear disutility from labour reflects a constant marginal value of leisure, in contrast to a decreasing marginal utility of consumption. Substituting one’s net income and total income, we have *c* – *βC* = (1 – *t*)*wl* + *B* – *βy*. A non-working^4^ individual has *l* = 0 and their utility only depends on *B* – *βy* ≥ 0. Thus, the utilities for the employed and non-working, denoted by *V* and *U*, are:

1 $$V = \frac{{1 + \varepsilon }}{\varepsilon } {[(1 - t)wl+B - \beta y]^{\frac{\varepsilon }{{1+\varepsilon }}}}$$

2 $$U =\frac{{1 + \varepsilon }}{\varepsilon } {(B - \beta y)^{\frac{\varepsilon }{{1+\varepsilon }}}}$$

*ε * (> 0) is elasticity of labour supply with respect to the net wage, (1 – *t*)*w*. (2) requires *B* – *βy* ≥ 0. In the case of *B* – *βy* < 0, we define *U* = 0. We will show later that this will not happen. In (1) and (2) total earnings impose a negative externality on everyone, –*βy*. When *β* approaches zero, there is no comparison. We consider the effects of a positive *β* and the policy response to its increase. From (1) we obtain the first-order condition of utility maximization, i.e., $\frac{{dV}}{{dl}}$ = 0 for a worker with wage *w*:

3 $$(1-t)^{{1+\varepsilon}}\left( {\frac{w}{A}} \right)^{{1 + \varepsilon }} = (1-t)wl + B-\beta y$$

From (3) we can solve for the optimal individual labour supply as:

4 $$l^* = {\text{ }}\left( {{\text{1 }}{-}t} \right)^{{\text{e}}} \frac{{w^{\varepsilon } }}{{A^{{1 + \varepsilon }} }} - \frac{{B - \beta y}}{{(1 - t)w}}$$

Given *B* – *βy* ≥ 0, the optimal labour supply increases with *w*. It decreases with *t* given *B* – *βy*. An individual chooses to work if and only if their utility *V* is higher than *U*. We define the *marginal* wage *m* (≥ 0) as the highest wage at which one chooses zero labour supply (non-employment). By definition, a marginal wage earner is indifferent between work and non-employment. From (4) with l* = 0, we have

5 $$(1-t)^{{1+\varepsilon}} \left( {\frac{m}{A}} \right)^{{1 + \varepsilon }} = B-\beta y$$

Given any *t* < 1, (5) implies that *m* > 0 if *B* > *βy*. As *B* = *ty*, it implies *t* > *β*, which will be justified below. Anyone with wage lower than or equal to *m* will choose non-employment, perhaps doing unpaid work at home and supported by the basic income. The non-employment rate is *F*(*m*), and the employment rate *E*(*m*) = 1 – *F*(*m*). Combining (5) and (3) we find earnings for a worker with *w > m* as:

6 $$wl^* = {\text{ }}(1-t)^\varepsilon\frac{{w^{{1 + \varepsilon }} - m^{{1 + \varepsilon }} }}{{A^{{1 + \varepsilon }} }}$$

Integrating (6) over all *w* > *m* yields total output *y* = $$\int_{m}^{b} {wl^*f(w)dw}$$. Let function *G*(*m*) $$\equiv \text { }$$$$\int_{m}^{b} {{w^{1+\varepsilon }}f(w)dw}$$. Given (6) and *E*(*m*) = 1 – *F*(*m*), we can write total output as:

7 $$y = (1-t)^\varepsilon \frac{{G(m) - m^{{1 + \varepsilon }} E(m)}}{{A^{{1 + \varepsilon }} }}$$

Given marginal wage *m*, total output falls with *t*. However, as *t* changes, *m* will be affected. Here we have three variables, *β*, *t* and *m*, consistent with optimal labour supply. *β* is exogenous, *t* is the choice variable and *m* is a state variable determined by *β* and *t*. In the next section we will show that concern for relative income will raise the optimal labour supply, reduce everyone’s utility and increase inequality.

## Impact of comparison

From (1) and (2) we see the direct effect of *β* on *V* and *U* given a fixed tax *t*. Moreover, indirectly it will stimulate labour supply in (4), thus raise total output *y* and basic income *B*, also raises the negative externality *βy*. To evaluate the total impact of *β*, we first express the utilities of the employed and non-employed in terms of *t* and *m*. Substituting (3) into (1), we get *V* = $\frac{{1 + \varepsilon }}{\varepsilon }$(1 – *t*)^ε^${(\frac{w}{A})^\varepsilon }$– *Al*. Substituting l* from (6) here, we find the maximised the utility V*. Similarly, substituting (5) into (2), we get the maximum U*.

8 $$V^*={\left(\frac{{1 - t}}{A}\right)^\varepsilon }\left( {\frac{{{w^\varepsilon }}}{\varepsilon }+\frac{{{m^{1+\varepsilon }}}}{w}} \right)$$

9 $$U^* = \frac{{1 + \varepsilon }}{\varepsilon }\left( {\frac{{1 - t}}{A}} \right)^{\varepsilon } m^\varepsilon$$

Though (8) and (9) do not contain *β* explicitly, V* and U* are affected by *β* through *m*. While V* always increases with the wage *w*, it is equal to U* when *w* = *m*. To simplify our analysis, we define a function *h*(*m*) as follows:

10 $$h(m) = 1 - \frac{{{m^{1+\varepsilon }}}}{{G(m)+{m^{1+\varepsilon }}F(m)}}$$

Obviously, 0 ≤ *h*(*m*) ≤ 1. For any *m* < *b*, *m*^1+ε^ < *G*(*m*) + *m*^1 + ε^*F*(*m*), so *h*(*m*) > 0. For any *m* > 0, *h*(*m*) < 1. As *G*’(*m*) = –*m*^1+ε^*f*(*m*), *h*(*m*) falls with *m* if (1 + *ε*)*m*^ε^[*G*(*m*) + *m*^1 + ε^*F*(*m*) – *m*^1+ε^] > 0, which is guaranteed since *G*(*m*) > [1 – *F*(*m*)]*m*^1+ε^. Hence, we have.

### Lemma 1

*Given any t and β*, $h^{\prime}\left( m \right)<0$.

Substituting *B* = *ty* and (7) into (5), we obtain (1 – *t*)*m*^1+ε^ = (*t* – *β*)[*G*(*m*) – *m*^1 + ε^*E*(*m*)]. As *t* – *β* = 1 – *β* – (1 – *t*), we get (1 – *t*)[*G*(*m*) + *m*^1 + ε^*F*(*m*)] = (1 – *β*)[*G*(*m*) – *m*^1 + ε^*E*(*m*)]. So, we obtain a simple relation:

11 $$1-t = (1-\beta)h\left( m \right)$$

Thus, *m* is an implicit function of *t* and *β*. When *t* = *β*, we have *h*(*m*) = 1, i.e., *m* = 0. If *m* > 0, *h*(*m*) < 1, then *t* > *β*. When *β* is close to 1, we must have *t* = 1. Then (6) implies that all labour supply will be zero. This is not an interesting case as no production takes place. We rule it out and assume that *β* is not close to 1.

From (11) we can see how *m* is affected by *β*. Given *t* we differentiate (11) with respect to *β* and obtain –*h*(*m*) + (1 – *β*)$$h^{\prime}(m)$$$$\frac{{\partial m}}{{\partial \beta }}$$ = 0. Hence $$\frac{{\partial m}}{{\partial \beta }}$$ = $$\frac{{h(m)}}{{(1 - \beta )h^{\prime}(m)}}$$ < 0 as $$h^{\prime}(m)$$ < 0.

### Lemma 2

*Given any t*, $$\frac{{\partial m}}{{\partial \beta }}$$* <* 0.

As *m* falls due to a larger *β*, employment rises, so stronger comparison raises labour supply on extensive margins. Given Lemma 2, we can look at the impact of *β* on labour supply on intensive margins. Given (6), a worker’s labour supply l* = $$\frac{{{{(1 - t)}^\varepsilon }}}{{{A^{1+\varepsilon }}}}$$(*w*^ε^ – $$\frac{{{m^{1+\varepsilon }}}}{w}$$). Since $$\frac{{\partial m}}{{\partial \beta }}$$* <* 0, we find$$\frac{{\partial l^*}}{{\partial \beta }}$$ = –$$\frac{{{{(1 - t)}^\varepsilon }}}{{{A^{1+\varepsilon }}}}$$(1 + ε)$$\frac{{{m^\varepsilon }}}{w}$$$$\frac{{\partial m}}{{\partial \beta }}$$ > 0. Hence stronger comparison always raises every worker’s labour supply on intensive margins. Moreover, as $$\frac{{\partial l^*}}{{\partial \beta }}$$ is inversely related to *w*, the lower wage earners are affected more.

### *Proposition* 1

**KUJ**: *Given any fixed tax, stronger comparison raises labour supply on both extensive and intensive margins but affects lower earners more.*

Since lower earners have lower labour supply in (4), a large KUJ effect implies a bigger proportional increase in work time for low earners, indicating an even more unequal impact. In the real world, higher earners normally adjust their work hours less because of fewer opportunities for part time work^5^. Our result points to a worse situation from a higher *β* for lower earners, as they suffer more KUJ effect than higher earners.

These different responses in labour supply to stronger comparison will lead to different utility losses. V*and U* incorporate the labour supply response, consequential changes in total output *y* and basic income *B*, and summarise overall effects through the marginal wage *m*. As $$\frac{{\partial m}}{{\partial \beta }}$$* <* 0, (8) and (9) show that both employed and unemployed are worse off with lower *m* due to a higher *β*. From (8) we obtain $$\frac{{\partial V^*}}{{\partial m}}$$ = (1 + *ε*)$${(\frac{{1 - t}}{A})^\varepsilon }$$$$\frac{{{m^\varepsilon }}}{w}$$ > 0 and this falls with *w*. From (9) we find $$\frac{{\partial U^*}}{{\partial m}}$$ = (1 + *ε*)$${(\frac{{1 - t}}{A})^\varepsilon }$$*m*^ε−1^ > 0 and bigger than $$\frac{{\partial V^*}}{{\partial m}}$$ since *w* > *m*. Hence lower earners are affected more and the unemployed most.

### *Proposition* 2

*Given any fixed tax, stronger comparison reduces everyone’s utility, but affect lower earners more and the non-employed most*.

Therefore we find a larger proportional welfare loss for the poor who suffer more utility loss from a lower initial level. This result is consistent with FitzRoy and Nolan (2016), but differs from Ulph (2014) who shows if everyone compares their income only with those with the same wage, a low paid worker will end up being worse off than the unemployed. The wellbeing of the unemployed being lowest seems more consistent with empirical evidence. Our finding indicates that comparison does not only hurt everyone, but also raises inequality by inflicting the greatest loss on the poorest. Hence a tax response should not only offset the inefficiency due to a negative externality but also reduce inequality.

## Tax response

Since stronger comparison increases KUJ and harms the unemployed most, one natural response is to follow a *maxi-min policy*, to maximize the utility of the worst-off, the unemployed, or equivalently, the lowest paid employed who share the same utility. Kanbur and Tuomala (2013) and FitzRoy and Nolan (2016) compare the optimal tax policy under maxi-min and utilitarian objectives. We first consider maxi-min as a special case of a more general welfare function. Although not a realistic policy option, the maxi-min tax can lead to a simple result to illustrate new insight compared with the previous literature.

We will examine the impact of a tax response through the marginal wage *m*. To do so we first see how *t* affects *m*. Given *β*, we differentiate both sides of (11) with respect to *t*, which leads to (1 – *β*)$$h^{\prime}(m)$$$$\frac{{\partial m}}{{\partial t}}$$ = − 1 which implies$$\frac{{\partial m}}{{\partial t}}$$ = − 1/(1 – *β*)$$h^{\prime}(m)$$ > 0. Hence, we have

### Lemma 3

*Given any β*, $$\frac{{\partial m}}{{\partial t}}$$* >* 0.

From (9) we see that U* increases with *m*(1 – *t*) = (1 – *β*)*mh*(*m*). Then for any *β*, to maximize U* is equivalent to maximizing *mh*(*m*), which cannot happen if *m* = 0. As we mentioned earlier, *m* > 0 implies *t* > *β*. With a general income distribution, the maxi-min tax rate cannot be equal to *β* as in Ljungqvist and Uhlig (2000).

We assume there is a unique *m*, denoted by m* that maximises *mh*(*m*), and hence U*. Surprisingly, this m* does not depend on *β*, as 1 – *β* is a multiplicative term. The maxi-min tax should ensure that the marginal wage equals m*. From (11) we have t* = 1 – (1 – *β*)*h*(m*). When *β* rises, t* thus increases, but *h*(m*) remains the same.

### *Proposition* 3

The maxi-min tax t* must be higher than β and maintain a marginal wage m*, which is ***independent ***of β, though t* increases with β.

Given a worker’s labour supply l* = $\frac{{{{(1 - t)}^\varepsilon }}}{{{A^{1+\varepsilon }}}}$(*w*^ε^ – $\frac{{{m^{1+\varepsilon }}}}{w}$) from (6), when the tax rises in response to stronger comparison and maintains the marginal wage, l* must fall, but more so for higher earners. Moreover, everyone’s utility will fall in the same proportion of (1 – *t*)^ε^ as shown in (8) and (9). Since higher wage earners have higher initial utility, they will lose more than those lower down the distribution, with the unemployed suffering the least, consistent with the social objective to help the poorest under a maxi-min objective.

However, the maxi-min policy is problematic because it ignores the welfare of the employed. Different from Corneo (2002), our maxi-min tax will hurt high earners and thus does not lead to a Pareto improvement. Since the employed form a majority in every society, this will reduce utilitarian welfare and political feasibility. Hence, we should consider a more plausible objective, to maximize a weighted average utility of the whole population.

We assign a weight *s*(*w*) to an individual with wage *w*. The weight for an unemployed person is *s*(*m*). The total weights sum to one, i.e., *s*(*m*)*F*(*m*) + $\int_{m}^{b} {f(w)s(w)dw}$ = 1. We assume that *s*(*w*) is non-increasing in *w*, so the poor have no less weight than the rich. Multiplying (8) and (9) by *s*(*w*) and *s*(*m*) respectively and summing over all individuals, we obtain our weighted average social welfare of the whole population, *SW* = *s*(*m*)*F*(*m*)U* + $\int_{m}^{b} {f(w)s(w)V^*dw}$. Substituting for U* and V* from (8) and (9) and for 1 – *t* by (11), our welfare function can be written as:

12 $$SW = \left[ {\frac{{(1 - \beta )h(m)}}{A}} \right]^{\varepsilon } \left[ {\frac{{1 + \varepsilon }}{\varepsilon }m^{\varepsilon} s\left( m \right)F\left( m \right) + \int_{m}^{b} {\left( {\frac{{w^{\varepsilon } }}{\varepsilon } + \frac{{m^{{1 + \varepsilon }} }}{w}} \right)f(w)s(w)dw} } \right]$$

This is a special case of the Bergson-Samuelson social welfare function, commonly used in the tax literature following Mirrlees (1971). To maximize (12), the optimal tax *t* and the corresponding marginal wage *m* will depend on weights given to the poor and the rich.

However, regardless of these weights, we can show that an optimal tax must be lower than t* and the marginal wage *m* lower than m*. If *m* > m*, a tax reduction will lower *m* towards m*. Thus, it will benefit the unemployed, also lead to higher net earnings (1 – *t*)*wl* and benefit all the employed. So, we could have a Pareto improvement and *m* > m* must be Pareto inefficient. Hence, we must have *m* ≤ m*. As $\frac{{\partial m}}{{\partial t}}$* >* 0, the corresponding optimal tax cannot exceed t*. If we have *s*(*w*) = 0 for all *w* > *m*, the ‘sum to one’ property of the weighting function implies *s*(*m*)*F*(*m*) = 1, so (12) reduces to maxi-min and the corresponding optimal tax is t*.

Moreover, (12) cannot be maximized if *m* = 0, which implies *h*(*m*) = 0. As we mentioned earlier, *m* > 0 implies *t* > *β* by (11). The intuition is clear. As *t* rises, *V* in (1) will rise if *y* – *wl* + (*t* – *β*)$\frac{{\partial y}}{{\partial t}}$ > 0. If *t* = *β*, it holds for any *wl* < *y*. Hence, it benefits all workers with less than average earnings including the unemployed^6^. Then our decreasing marginal utility of consumption guarantees a higher *SW* with a non-increasing weight for higher earners. So, *t* = *β* cannot be optimal. Obviously, this does not apply if the population is represented by a single agent, or if all agents have the same marginal utility as in the earlier literature.

### Proposition 4

Given a general income distribution and a welfare function with non-increasing weight to higher earners, an optimal tax t must satisfy t* ≥ *t* > β and m* > *m >* 0.

Our result differs from Ljungqvist and Uhlig (2000) and the subsequent literature with a representative agent. However, given t* ≥ *t* > *β*, we still do not know the precise optimal tax which will depend on the income distribution and social welfare function, especially the relative weight assigned to the poor and the rich. Finding such an optimal tax is difficult since the policy maker may not have the above information as well as income comparison *β*. In fact, a tax policy would not be useful if it is very sensitive to unobservable and unreliable information. We need a practical method to avoid this difficulty.

To find the optimal *t*, we see that *β* is again separable from the rest of the function (12), which only depends on *m*. The optimal *m* maximizing (12) must be independent of *β*. For simplicity we again assume there is a unique maximum $\bar {m}$. While the optimal $\bar {m}$ is independent of *β*, similar to m* before, the corresponding optimal tax, $\bar {t}$= 1 – (1 – *β*)*h*($\bar {m}$) from (11), clearly positively depends on *β*.

### *Proposition* 5

*To maximize a weighted average utility, the optimal tax should maintain a constant marginal wage *$\bar {m}$ (< m*), *again independent of β*.

When *β* is close to 1, the optimal tax $\bar {t}$ will be nearly 100% as found by Boskin and Sheshinski (1978). Nevertheless, for a reasonable *β*, the optimal $\bar {m}$ remains fixed even though *β* rises, because a higher tax just cancels out the impact of KUJ on extensive margins. Moreover, given *β*, $\bar {t}$also depends on *s*(*w*). When more weight is given to high earners, $\bar {m}$ tends to be lower, which implies higher *h*($\bar {m}$). Then the optimal tax rate will be lower.

As *β* is not observable and $\bar {m}$ depends on the weighting function *s*(*w*), the optimal tax $\bar {t}$ seems to be intractable, so as is common in economics, the ideal solutions may not be implementable. However, if the original employment is at its optimal level before comparison rises, a reasonable policy is to simply adjust taxes to keep the (voluntary) employment level unchanged when *β* rises. The level of employment reflects our social preference. The higher is employment, the higher the weight for low-wage earners and the unemployed. If the policy maker changes their preference, a new target level of employment can be chosen accordingly.

While a higher *β* raises individual labour supply, our optimal tax response to keep *m* = $\bar {m}$ reverses this impact. As (6) implies l* = $\frac{{{{(1 - t)}^\varepsilon }}}{{{A^{1+\varepsilon }}}}$(*w*^ε^ – $\frac{{{m^{1+\varepsilon }}}}{w}$), the higher optimal $\bar {t}$ to maintain fixed $\bar {m}$ will reduce labour supply on intensive margins. The tax effect dominates that of KUJ due to stronger comparison, so we have:

### *Proposition* 6

***reversing KUJ***: *The tax response to stronger comparison will reduce labour supply on intensive margins and reverse KUJ.*

Different again from Corneo (2002), whose optimal progressive taxes just correct KUJ, our policy not only maintains labour supply at extensive margins, but reverses KUJ on intensive margins. It can be explained intuitively. As comparison creates a negative externality –*βy*, a bigger *β* raises the negative impact of *y*. Hence the social planner should reduce *y* below its original level. To do so, the tax should reduce labour supply l* below its original value, i.e. reversing KUJ. The idea is to put less value on consumption in favour of equality. It will shorten work time and allow more leisure. If implemented, this policy would help reverse persistent long working hours and restore work-life balance.

Finally, we evaluate the net effect on everyone’s utility, given our optimal tax response. Substituting (11) into (8), we get V* = ${[\frac{{(1 - \beta )h(m)}}{A}]^\varepsilon }$($\frac{{{w^\varepsilon }}}{\varepsilon }$ + $\frac{{{m^{1+\varepsilon }}}}{w}$). When *β* rises, the tax response keeps *m* and *h*(*m*) fixed, everyone’s utility falls as (1 – *β*)^ε^ does. It implies bigger losses for higher earners since $\frac{{{w^\varepsilon }}}{\varepsilon }$ + $\frac{{{m^{1+\varepsilon }}}}{w}$ rises with *w* from its minimum at *m*.

### *Proposition* 7

*With an optimal tax policy, everyone is still worse off when comparison becomes stronger, but lower earners* (*the non-employed*) *suffer less* (*least*).

This is opposite to the utility loss without tax responses (see Proposition 2). Hence the tax response must benefit low earners and the unemployed, although the overall impact of a higher *β* is still negative for everyone. Furthermore, as everyone’s utility falls by the same proportion, our tax policy reduces the absolute gap between the rich and poor and leads to lower inequality.

This policy essentially redistributes income in response to stronger comparison to reduce inequality, which would increase otherwise. Thus, it not only resists the negative shock, but also turns it into a positive change in terms of equality. While the tax rise reduces total output, the loss is compensated by a higher level of weighted average utility than the case without the tax response. This trade-off between lower total output and higher equality seems to be justified under a general social welfare function.

## Conclusion

Full-time working hours have not declined compared to early this century in the UK and since the 1980s in the US. Besides increased employer market power and declining union bargaining power, comparison or relative income concern is likely to be an important factor, encouraged by social media and rising inequality. We find that the optimal tax response to stronger comparison should maintain constant employment, though the tax itself rises with comparison. The tax response will dominate the KUJ effect and *reduce* labour supply on intensive margins.

Our policy recommendation to reverse KUJ is also consistent with recent calls and experiments to cut working time to four days a week. On the other hand, our tax policy does not reduce labour market participation and employment at the extensive margin. The information requirement is minimal in comparison to the optimal tax literature. The main message is that taxes need to be increased in response to stronger comparison not only to reduce inefficiency, but also inequality. The additional benefits of reducing inequality have not been captured in previous models.

Moreover, our analysis demonstrates benefits of a basic income (demogrant) as assumed here and widely used in the literature. At the moment, such an unconditional basic income only exists in special circumstances in the real world, e.g., Covid-19 relief for most Americans. However, discussion of the merit of a basic income has become increasingly popular in many countries – in some instances, leading to limited trialling.

Clearly, further research is needed to see how the tax policy holds up in a more general setting. The current undesirable work-life balance requires other policy reforms. For instance, market imperfections imply that stronger regulation to reduce employer power is also needed, as well as employee participation in management and more effective union bargaining power to curb the demand side pressure and widespread abuses such as unpaid overtime.

## Electronic supplementary material

Below is the link to the electronic supplementary material.

Supplementary Material 1

## Data Availability

Not applicable.

## Footnotes

1 More recent empirical contributions include Ferrer-i-Carbonell (2005), Senik (2009), Dohmen et al. (2011), Card et al. (2012), FitzRoy et al. (2014), FitzRoy and Nolan (2018).

2 With two types of agents Aronsson and Johansson-Stenman (2013, 2015 and 2018) obtain similar results in other settings.

3 We thank an anonymous referee for the suggestion of this formulation.

4 These individuals may be voluntarily unemployed, or doing unpaid housework (alongside being non-employed in the market), so we use ‘non-working’, ‘unemployed’ and ‘non-employed’ as interchangeable terms. Involuntary unemployment could be included but would not provide additional insight.

5 Except in the Netherlands, with the best work-life balance and flexibility in the OECD, while labour market flexibility is likely to increase everywhere in the wake of the Covid-19 pandemic. Particularly in the UK and US, high earners have often been required to work long hours.

6 As the median income is always lower than the mean, we know that the tax rate must be higher than *β* to maximize the utility of the poor majority.
