# Supplementary material for: Higher tax and less work: reverse “Keep up with the Joneses” and rising inequality
Source: J Econ (Vienna). 2023 Mar 29;139(3):177–90. doi: 10.1007/s00712-023-00821-2 (PMC10050818; doi:10.1007/s00712-023-00821-2)
Supplement: Supplementary file 1 — Supplementary Material 1 [file 712_2023_821_MOESM1_ESM.docx]

Dear Professor Giacomo Corneo.

Editor-in-Chief, Journal of Economics,

Thank you for agreeing to consider a major revision of our paper “Higher Tax and Less Work” to be considered by JoE. Please note that we are proposing an adjustment to the part of the paper title after the colon – so the overall title adjusts from “Higher Tax and Less Work: How not to Keep up with the Joneses” to “Higher Tax and Less Work: Reverse “Keep up with the Joneses” and Rising Inequality”.

We have made changes responding to all comments by the two referees.

Dear Referee 1:

We thank you again for your constructive suggestions which have been adopted in our current version and are explained below.

1. “*In the intro, it takes somewhat long until it is written what is done in the paper: The “In this paper” paragraph is the last one of the intro and does not add much to what is written in the abstract. I would bring the description of model earlier in the intro and also write more on what the main findings are, and in what way they contribute to the existing literature*.”

Following this suggestion, we now start with a short summary of our paper at the beginning, and shortened the literature review section, and in the end of the introduction we write a more detailed section to explain our main findings and why they contribute to the literature. In particular we explain why inequality is crucial with heterogeneous agents and implies the optimal tax higher than *β*, instead of equal as in the previous literature. Moreover, since this tax is sensitive to income distributions and welfare functions as well as unobservable *β*, we need a practical method independent of this unrealistic information.

2. “*For me the modelling of status is still a bit ad hoc. Status is modelled by assuming that the average gross income enters negatively utility. It should be discussed more what the implication of this modelling for the results is. Why assume that the gross wage negatively enters utility, and not the net wage*?”

As suggested by Referee 2, we now cite Johansson-Stenman et al. (2002) which justifies this particular formulation of comparison. Let *c* be one’s consumption and *C* average consumption. We write our sub-utility as a function of two parts of consumption, where (1 – *β*)*c* depends on one’s own consumption and *β*(*c* – *C*) is the status effect dependent on relative consumption. This function *u*[(1–*β*)*c* + *β*(*c*–*C*)] turns into *u*(*c* – *βC*) as in Johansson-Stenman et al. (2002) and also identical to Ljungqvist and Uhlig (2000). The implication is added in the paper. We also explain that *c* is equal to one’s net income and *C* average income which is also average net income. Therefore, our comparison, *β*(*c* – *C*), is indeed between one’s net income or consumption and average consumption of the population or its net income.

3. “*The assumption B – βy > 0 deserves more discussion. Wouldn’t one normally assume that the benefit level of the poor is smaller than average income y? So, I impose that status concerns are not overly strong to make the assumption valid, which is fine, but should be discussed more*.”

Yes, indeed we assume *B* < *y*. As *B* = *ty*, it implies *t* < 1 (tax is lower than 100%). Then *B* – *βy* > 0 holds if and only if *t* > *β*. So *β* cannot be close to 1, i.e., one’s utility still depends on his own consumption, not only the status or position. We add this explanation to the paper.

4. “*Lemma 2 is not clear to me. It states that “A reasonable social welfare function requires t > β”, but at this point a social welfare function is not defined yet. Is it meant that the optimal tax in this model will be larger than β*?”

Yes, we meant that *t > β* under “any social welfare function” which “assigns at least equal weight to lower income earners than higher ones”, including maxi-min and utilitarian. However, we recognize that it was not clearly explained, and now formally show *t* > *β* after defining the welfare function (12). Moreover, the original Lemma 2 is incorporated into the modified Proposition 4 for clearer presentation.

5. “*I would heavily streamline the paper. Also the propositions jump a bit between optimal tax results and comparative statics of status concerns on labor supply and welfare*.”

Thank you for this suggestion and we tried to streamline the paper. We move any discussion regarding the optimal tax from Section 3 to Section 4, where the impact of comparison and the tax are discussed respectively. Moreover, we split the original Lemma 1 into two for gradual progression and easier understanding. The original Proposition 4 is modified and combined with the original Lemma 2 to become new Proposition 4, which gives the range of the optimal tax under a general welfare function. We think it connects the text more smoothly.

6. Thank you for pointing out our typo of “Proposition 2”, which should be Lemma 2 as you suggested. Also, we add the definition of *B* when it is first introduced.

In addition, follow the other referee’s suggestion, we modified our model to be more consistent with Ljungqvist and Uhlig (2000) by adding a constant coefficient *A* in the front of labour supply in the utility function. Finally, we made minor changes in the paper for better and more accurate presentation.

If you have more comments and suggestions, we will be happy to take them into consideration in further revisions.

Dear Referee 2:

We thank you again for your constructive suggestions which have been adopted in our current version and are explained below.

1. “*The motivation of quasi-linearity in leisure is still not satisfying* *in the paper*.”

We cite Ljungqvist and Uhlig (2000) which uses the same quasi-linear utility function. We also add an extra justification for the linearity of the utility loss from labour, i.e., a constant marginal value of leisure in contrast to decreasing marginal utility of consumption.

Moreover, to be fully consistent with Ljungqvist and Uhlig (2000), we add a constant coefficient *A* in the front of labour supply *l* in the utility function. We have adjusted the mathematical expressions throughout the paper accordingly.

2. “*The formulation of the basic model of social comparisons could easily be formulated in a much clearer way (this is related also to a point by the other reviewer)*.”

We completely agree with your suggestion and write our sub-utility function in terms of consumption, i.e., *u*[(1–*β*)*c* + *β*(*c*–*C*)] = *u*(*c* – *βC*). Thanks for your information we now cite Johansson-Stenman et al. (2002) to justify this particular formulation regarding income comparison. Consequently, we adopt your interpretation of the two terms in the sub-utility function so that our original “unclear effective consumption” has been dropped.

3. Following your suggestion, we changed our notation for labor supply from x to l.

In addition, following the other referee’s suggestion we tried to streamline our paper. We move any discussion on the optimal tax from Section 3 to Section 4, where the impact of comparison and the tax are discussed respectively. The original Lemma 1 is split into two for gradual progression and easier understanding. The original Proposition 4 is modified and combined with the original Lemma 2 to become new Proposition 4, which gives the range of the optimal tax under a general welfare function. We think it connects the text more smoothly. Finally, we made minor changes in the paper for better and more accurate presentation.

If you have more comments and suggestions, we will be happy to take them into consideration in further revisions.
